# Supplementary material for: The AID study: protocol for a randomised controlled trial of intrauterine insemination in the natural cycle compared with intracervical insemination in the natural cycle
Source: BMJ Open. 2019 Jul 23;9(7):e026065. doi: 10.1136/bmjopen-2018-026065 (PMC6661593; doi:10.1136/bmjopen-2018-026065)
Supplement: Author's manuscript [file bmjopen-2018-026065.draft_revisions.pdf]

BMJ Open

BMJ Open is committed to open peer review. As part of this commitment we make the peer review history of every article we publish publicly available.

When an article is published we post the peer reviewers' comments and the authors' responses online. We also post the versions of the paper that were used during peer review. These are the versions that the peer review comments apply to.

The versions of the paper that follow are the versions that were submitted during the peer review process. They are not the versions of record or the final published versions. They should not be cited or distributed as the published version of this manuscript.

BMJ Open is an open access journal and the full, final, typeset and author-corrected version of record of the manuscript is available on our site with no access controls, subscription charges or pay-per-view fees (http://bmjopen.bmj.com).

If you have any questions on BMJ Open's open peer review process please email info.bmjopen@bmj.com

BMJ Open

Artificial insemination with donor sperm: a randomised controlled trial of intrauterine insemination in the natural cycle compared to intracervical insemination in the natural cycle.

Journal:	*BMJ Open*
Manuscript ID	bmjopen-2018-026065
Article Type:	Protocol
Date Submitted by the Author:	20-Aug-2018
Complete List of Authors:	Kop, Petronella; Center for Reproductive Medicine<br>van Wely, Madelon; Academic Medical Center, Obstetrics and Gynaecology<br>Willem Mol, Ben; Monash University Central Clinical School<br>Bernardus, R.E.; Nij Barrahus<br>Brucker, M.; Universitair Ziekenhuis Brussel<br>Janssens, Pim; Rijnstate Hospital, Clinical Chemistry and Haematology<br>Nap, Annemiek; Rijnstate<br>Cohlen, B.J.<br>Pieters, Jacqueline; VivaNeo Medisch Centrum Kinderwens<br>Repping, S.<br>van der Veen, F.<br>Mochtar, Monique
Keywords:	Reproductive medicine < GYNAECOLOGY, intrauterine insemination, intracervical insemination, donor sperm

SCHOLARONE™ Manuscripts

Artificial insemination with donor sperm: a randomised controlled trial of intrauterine insemination in the natural cycle compared to intracervical insemination in the natural cycle.

PAL Kop[1], M van Wely[1], BW Mol[2], RE Bernardus[3], M de Brucker[4], PMW Janssens[5], A Nap[5] , BJ Cohlen[6], J.J.P.M. Pieters [7], S Repping[1], F van der Veen[1] and MH Mochtar[1].

[1] Center for Reproductive Medicine, Academic Medical Center, Amsterdam, The Netherlands
[2] Department of Obstetrics and Gynaecology, Monash University, Melbourne, Australia
[3] Nij Barrahus, Wolvega, The Netherlands
[4] University Hospital Brussels, Brussels, Belgium
[5] Rijnstate, Arnhem, The Netherlands
[6] Isala Zwolle, Zwolle, The Netherlands
[7] VinaNeo Medisch Centrum Kinderwens, Leiderdorp, The Netherlands

For peer review only

ABSTRACT

Introduction At present, studies comparing intrauterine insemination in the natural cycle versus intracervical insemination in the natural cycle in women undergoing artificial insemination with donor sperm are scarce.

Methods and analysis We perform a randomised controlled non-inferiority trial among five secondary and tertiary fertility clinics in the Netherlands and one tertiary fertility clinic in Belgium. Women eligible for artificial insemination with donor sperm are included. We perform six cycles of artificial insemination with donor sperm within a time horizon of eight months comparing intrauterine insemination in the natural

For peer review only - http://bmjopen.bmj.com/site/about/guidelines.xhtml

cycle with intracervical insemination in the natural cycle. The primary outcome is ongoing pregnancy leading to live birth conceived within eight months after randomisation. Secondary outcomes are clinical pregnancy rate, miscarriage rate, multiple pregnancy rate, pregnancy complications (preterm birth, birth weight < 2500 gram, pregnancy induced hypertension, (pre-) eclampsia, HELLP), time to ongoing pregnancy, direct and indirect costs. To demonstrate the non-inferiority of ICI with a margin of 12%, we need 208 women per arm.

Ethics and dissemination The study has been approved by the Medical Ethical Committee of the Academic Medical Centre and from the Dutch Central Committee on Research involving Human Subjects (47330-018-13). Results will be disseminated through peer-reviewed publications and presentations at international scientific meetings.

Trial registration number NTR4462.

Strengths and limitations of this study

- This is a multicentre randomised controlled trial
- We aim to give cumulative ongoing pregnancy rates
- The study is not blinded, which can be a limitation

BACKGROUND

Artificial insemination with donor sperm (AID) may be performed in heterosexual couples for medical reasons or to assist lesbian couples or single women to achieve pregnancy. Medical reasons for AID include obstructive and non-obstructive azoospermia, severely impaired semen quality in couples who do not wish to undergo or were not successful with ICSI, severe rhesus iso-immunization,

For peer review only - http://bmjopen.bmj.com/site/about/guidelines.xhtml

prevention of vertical transmission of a genetic defect or prevention of transmission of human immunodeficiency virus (HIV) in couples who do not wish to undergo semen washing. [1, 2] Women can be inseminated via the intrauterine (IUI) or the intracervical (ICI) route with or without ovarian stimulation.

A systematic review, in women who started AID, reported intrauterine insemination with ovarian stimulation (IUI-OS) to be more effective than intracervical insemination with ovarian stimulation (ICI-OS) in terms of live birth rate (OR 2.55 95% 0.72-8.96, 1 RCT, n=43) and clinical pregnancy rate (OR 2.83, 95% CI 1.38 to 5.78, 2 RCTs, n=131) at the cost of a higher multiple pregnancy rate (OR 2.77, 95% CI 1.00 to 7.69, 2 RCTs, n=131). In women who started AID in the natural cycle, IUI resulted in similar live birth rates as ICI in the natural cycle (OR 3.24, 95% 0.12-87.13, 1 RCT, n=26) but in higher clinical pregnancy rates in (OR 6.18, 95% CI 1.91-20.03, 2RCTs, n=76). There were no multiple pregnancies. [3]

Multiple pregnancies are associated with increased perinatal and maternal morbidity and perinatal mortality and are regarded as an adverse treatment outcome that is no longer acceptable and should be prevented. [4-6]

Therefore, the single existing guideline on artificial insemination with donor sperm, published by The Royal College of Obstetrics and Gynaecology (RCOG), recommends six cycles of IUI in the natural cycle. [1]

In 2012 we performed a retrospective nationwide cohort study among eight sperm banks in the Netherlands on IUI and ICI in the natural cycle. We included 1843 women of whom 1163 underwent 4269 cycles of IUI in the natural cycle and 680 underwent 2345 cycles of ICI in the natural cycle. Cumulative ongoing pregnancy rates over six treatment cycles were 40.5% for IUI and 37.9% for ICI, resulting in a hazard ratio (HR) of 1.02 (95% CI 0.84-1.23) for IUI versus ICI. [7]

For peer review only - http://bmjopen.bmj.com/site/about/guidelines.xhtml

The costs for IUI have been estimated to be four times higher than ICI, mostly because of the sperm processing inherent to IUI. [8]

Since evidence that underpins the superiority of IUI in the natural cycle over ICI in the natural cycle is scarce and IUI may generate higher costs than ICI for no increase in pregnancies, we propose a multicenter trial comparing IUI and ICI in the natural cycle with a cost effectiveness analysis alongside the trial.

OBJECTIVE

To compare the effectiveness of six cycles of intrauterine insemination in the natural cycle compared with six cycles of intracervical insemination in the natural cycle with cryopreserved donor sperm within a time horizon of eight months.

METHODS

Study design

This study is a non-blinded, multicentre, non-inferiority randomised controlled trial among five secondary and tertiary fertility clinics in the Netherlands and one tertiary fertility clinic in Belgium. Recruitment started on 3 June 2014.

We expect to complete recruitment on 1 January 2020.

Study population

Inclusion criteria

We will study women between 18 and 43 years with a regular cycle, be it spontaneously or after ovulation induction and with an indication for AID.

Indications for AID are medical reasons like obstructive and non-obstructive azoospermia, severely impaired semen quality in couples who do not wish to

For peer review only - http://bmjopen.bmj.com/site/about/guidelines.xhtml

undergo or were not successful with intracytoplasmatic sperm injection (ICSI), severe rhesus iso-immunization, prevention of vertical transmission of a genetic defect or prevention of transmission of human immunodeficiency virus in couples who do not wish to undergo semen washing, In addition, lesbian couples or single women who apply for AID are eligible to participate.

Exclusion criteria

Women with known double-sided tubal pathology, irregular menstrual cycles, in vitro fertilisation (IVF) or IUI in their history will not be eligible.

Informed consent procedure

Women eligible for participation in the study are invited for additional counselling by a research nurse to ensure that they are fully informed on the nature of the study by means of both oral and written information. Women who agree to participate are asked to sign a written informed consent of which they receive a copy.

Randomisation

Randomisation is performed by accessing a web-based data system that is used for randomisation in clinical trials and will be performed centrally with the use of a permuted-block-design with randomly selected block sizes that vary between two, four and six.

Couples are randomly allocated to either six cycles of IUI in the natural cycle or six cycles of ICI in the natural cycle.

Interventions

For peer review only - http://bmjopen.bmj.com/site/about/guidelines.xhtml

In the IUI cycles, ovulation detection is performed with LH tests or by transvaginal sonography, depending on the local setting. In case of monitoring with urinary LH tests, women test their urine once per day, starting on an individually calculated cycle day based on their basal body temperature chart. Women are inseminated with processed semen the day after the endogenous LH surge has been detected in the urine sample. Serum LH measurements are performed from day 11 of the menstrual cycle onwards and women are inseminated one day after the serum LH rise.[9]

In case follicular growth is monitored by transvaginal sonography, timing of insemination can be performed by ovulation triggering or by measuring serum LH levels. In case of ovulation triggering human chorionic gonadotropin (Pregnyl, Organon, Oss, The Netherlands) is administered when the dominant follicle has a diameter of at least 16mm. Women are inseminated 36 to 40 hours thereafter. The semen may be processed in two ways. One way is to process the semen against a density gradient centrifugation and/or a washing step with culture medium according to local laboratory protocol after thawing of the –unprocessed- cryopreserved semen and the second way is to process the semen against a density gradient centrifugation and/or washing step with culture medium according to local laboratory protocol before freezing of the semen.

In the ICI cycles, ovulation detection is in the same way as in the IUI arm and insemination is also timed identical to the IUI arm. Women are inseminated with unprocessed semen once per cycle, and preferably by a cervical cap. [6]

Before starting a new treatment cycle with IUI or ICI, women undergo a pregnancy test (hCG measurement in serum or urine). If the woman is not pregnant the next treatment cycle is started according to protocol. In case of a positive pregnancy test women are monitored by transvaginal sonography. Monitoring will take place at five

For peer review only - http://bmjopen.bmj.com/site/about/guidelines.xhtml

to nine weeks of amenorrhea to check whether an intrauterine gestational sac is present, i.e. a clinical pregnancy.

Subsequently, monitoring takes place at 11 to 12 weeks amenorrhea to register the presence of an intrauterine gestational sac with foetal heartbeat, i.e. an ongoing pregnancy.

Follow up

Pregnant women undergo an ultrasound at 7 and 11 weeks of gestation to classify the pregnancy as clinical or ongoing singleton or multiple pregnancy. Women are contacted by telephone to enquire on the course of pregnancy, delivery and the health of the child. Detailed information on maternal complications will be obtained from the responsible obstetrician or midwife. When data on the health of the child are insufficient, child health centres and/or paediatricians are contacted for specific information. Presumably, not all women complete the eight months of treatment. Couples who drop-out will largely represent normal patient flow. In both arms, women will be treated until ongoing pregnancy occurs within a time horizon of eight months. We aim to keep track of all couples who drop-out and to document the reason for the drop-out.

Patient and Public involvement

The Dutch patient organisation for patients with fertility problems (Freya) supports the study. When the study is finished we will request Freya to organise an informal meeting during which fertility experts from our study group will present information on the treatment options. We will ask Freya to publish an easy to read summary of the results on their homepage. The data will thus become available for the public. Study

For peer review only

For peer review only - http://bmjopen.bmj.com/site/about/guidelines.xhtml

participants are asked if they want to be informed about the results of the study during the informed consent procedure.

OUTCOME MEASURES

Primary outcome measure

The primary outcome is ongoing pregnancy leading to live birth. Ongoing pregnancy is defined as a positive heartbeat at 12 weeks of gestation. Only ongoing pregnancies that occur within the first eight months after randomisation count for assessment of the primary endpoint.

Secondary outcome measures

Secondary outcomes are clinical pregnancy (any registered heartbeat at ultrasound), miscarriage (registered heartbeat before 12 weeks of gestation) and multiple pregnancy (registered heartbeat of at least two foetuses at 12 weeks of gestation) and ectopic pregnancy, time to ongoing pregnancy leading to live birth.

We will register the following pregnancy outcomes: preterm birth < 37 weeks, iatrogenic preterm birth, spontaneous preterm birth, time to delivery, mean birth weight, birth weight < 2.500 gram, growth restriction defined as birthweight < 10th percentile, pregnancy Induced Hypertension (PIH), pre-eclampsia, HELLP-syndrome and direct and indirect costs.

Power calculation

The study is designed as a non-inferiority trial. We assume that six cycles can be completed within a fixed time horizon of eight months. Assuming a live birth rate of

For peer review only - http://bmjopen.bmj.com/site/about/guidelines.xhtml

40% after six cycles of IUI, we need 208 women per arm with an alpha of 0.05, and a beta of 0.80 to demonstrate the non-inferiority of ICI with a margin of 12%.[7] The margin of 12% are the differences shown in literature between IUI and ICI. [3]

Data analysis

We will compare baseline measurements female age, BMI, indication for AID, previous assisted reproductive treatments, previous pregnancies, parity, intoxications and referral status.

The analysis of all outcomes is on an intention-to-treat basis. For ongoing pregnancy we will test non-inferiority on basis of the absolute risk difference with the absolute margin of 12%. We will subsequently estimate differences in the primary and secondary outcomes as relative risks with 95% CI. We will also report p values. We will construct Kaplan-Meier survival curves for time to ongoing pregnancy. For continuous variables, we examine the distribution of the observations, and if normally distributed, we summarize them as means with SDs. If they are not normally distributed, medians and IQRs are reported. For dichotomous data, we provide proportions (or percentages). In addition to the baseline and outcome data, we also summarize the recruitment numbers, those lost to follow-up, protocol violations and other relevant data. We will analyze a maximum of six cycles of IUI or ICI performed within a time horizon of eight months after randomisation.

Economic evaluation

We will perform an economic analysis from a healthcare perspective alongside the clinical trial. We make a distinction between direct costs (costs like medical interventions and other healthcare costs like medical appliances) and indirect costs

For peer review only - http://bmjopen.bmj.com/site/about/guidelines.xhtml

(costs of productivity loss or time loss costs). We will analyse a cost-minimization or cost-effectiveness analysis depending on the outcome of ongoing pregnancy rates in both groups. We present the cost-effectiveness of each strategy as cost per ongoing pregnancy and costs per live birth. We explore the robustness of the results for various assumptions and parameter estimates in sensitivity analysis outcomes and we express these in incremental cost-effectiveness ratio graphs and cost-effectiveness acceptability curves. The economic evaluation will be reported in a separate paper.

DISCUSSION

In women who start AID, there is uncertainty about the most cost effective treatment modality. IUI-OS leads to higher clinical pregnancy rates compared to ICI-OS, but the incidence of multiple pregnancies with the addition of OS is substantial and hardly acceptable.[3] Studies on IUI and ICI in the natural cycle report no differences in live birth rate between IUI and ICI in natural cycles, but higher clinical pregnancy rates for IUI, without any multiple pregnancies. Therefore, the NICE guideline recommends IUI in the natural cycle for the first six cycles. [1] Since IUI compared to ICI in natural cycles shows no difference in live birth rate, IUI may generate higher costs than ICI for no increase in live birth rates. There are no large randomised controlled trials that addresses this issue by comparing IUI in the natural cycle with ICI in the natural cycle in women who start with AID.

ETHICS AND DISSEMINATION

The study has been approved by the Medical Ethical Committee of the Academic Medical Centre and from the Dutch Central Committee on Research involving Human

For peer review only - http://bmjopen.bmj.com/site/about/guidelines.xhtml

Subjects (47330-018-13). The boards of the participating hospitals approved the study. Results will be disseminated through peer-reviewed publications and presentations at international scientific meeting

For peer review only

For peer review only - http://bmjopen.bmj.com/site/about/guidelines.xhtml

References

1. *National institute for Health and Care Excellence (NICE). Fertility: Assessment and treatment for people with fertility problems*. 2013.
2. Practice Committee of American Society for Reproductive, M. and T. Practice Committee of Society for Assisted Reproductive, *Recommendations for gamete and embryo donation: a committee opinion.* Fertil Steril, 2013. **99**(1): p. 47-62.
3. Kop, P.A., et al., *Intrauterine insemination versus intracervical insemination in donor sperm treatment.* Cochrane Database Syst Rev, 2018. **1**: p. CD000317.
4. Cohlen, B., et al., *Multiple pregnancy after assisted reproduction.* Lancet, 2005. **366**(9484): p. 452-3; author reply 453-4.
5. Fauser, B.C., P. Devroey, and N.S. Macklon, *Multiple birth resulting from ovarian stimulation for subfertility treatment.* Lancet, 2005. **365**(9473): p. 1807-16.
6. Nyboe Andersen, A., et al., *Assisted reproductive technology in Europe, 2000. Results generated from European registers by ESHRE.* Hum Reprod, 2004. **19**(3): p. 490-503.
7. Kop, P.A., et al., *Intrauterine insemination or intracervical insemination with cryopreserved donor sperm in the natural cycle: a cohort study.* Human Reproduction, 2015. **30**(3): p. 603-7.
8. *Nederlandse zorgautoriteit*. 2012; http://www.nza.nl/zorgonderwerpen/zorgonderwerpen/ziekenhuiszorg/veelgesteldevragen/veelgestelde-vragen-dbc-dot/,].
9. Blockeel, C., et al., *Should an intrauterine insemination with donor semen be performed 1 or 2 days after the spontaneous LH rise? A prospective RCT.* Human Reproduction, 2014. **29**(4): p. 697-703.

Funding statement

This trial will receive funding from the Netherlands Organization for Health Research and Development (ZonMw project number 837002407)). There is no role in the study for ZonMw.

Author's contributions

PALK is responsible for the overall logistical aspects of the trial and drafted the paper. MvW, MHM, FvdV, SR and BWM designed the trial and were responsible for the development of the protocol. PALK, REB, MB, PMWJ, AN, BJC, JJPMP, MvW, MHM, SR, BMW and FvdV contributed to the protocol included patients and approved the final version of the paper.

For peer review only - http://bmjopen.bmj.com/site/about/guidelines.xhtml

Competing interest: None declared

For peer review only

For peer review only - http://bmjopen.bmj.com/site/about/guidelines.xhtml

SPIRIT

STANDARD PROTOCOL ITEMS: RECOMMENDATIONS FOR INTERVENTIONAL TRIALS

SPIRIT 2013 Checklist: Recommended items to address in a clinical trial protocol and related documents*

Section/item	Item No	Description
**Administrative information**		
Title	1	Descriptive title identifying the study design, population, interventions, and, if applicable, trial acronym<br>L2
Trial registration	2a	Trial identifier and registry name. If not yet registered, name of intended registry<br>L39
	2b	All items from the World Health Organization Trial Registration Data Set<br>L39
Protocol version	3	Date and version identifier<br>Footnote
Funding	4	Sources and types of financial, material, and other support<br>L244-246
Roles and responsibilities	5a	Names, affiliations, and roles of protocol contributors<br>Footnotes L248-252
	5b	Name and contact information for the trial sponsor<br>L244-246
	5c	Role of study sponsor and funders, if any, in study design; collection, management, analysis, and interpretation of data; writing of the report; and the decision to submit the report for publication, including whether they will have ultimate authority over any of these activities<br>L246
	5d	Composition, roles, and responsibilities of the coordinating centre, steering committee, endpoint adjudication committee, data management team, and other individuals or groups overseeing the trial, if applicable (see Item 21a for data monitoring committee)<br>L90-94
**Introduction**		

For peer review only

Background and rationale	6a	Description of research question and justification for undertaking the trial, including summary of relevant studies (published and unpublished) examining benefits and harms for each intervention<br>L47-76
	6b	Explanation for choice of comparators<br>L79- 82
Objectives	7	Specific objectives or hypotheses<br>L84-87
Trial design	8	Description of trial design including type of trial (eg, parallel group, crossover, factorial, single group), allocation ratio, and framework (eg, superiority, equivalence, noninferiority, exploratory)<br>L91- 94

Methods: Participants, interventions, and outcomes

Study setting	9	Description of study settings (eg, community clinic, academic hospital) and list of countries where data will be collected. Reference to where list of study sites can be obtained<br>L91-94
Eligibility criteria	10	Inclusion and exclusion criteria for participants. If applicable, eligibility criteria for study centres and individuals who will perform the interventions (eg, surgeons, psychotherapists)<br>L97-110
Interventions	11a	Interventions for each group with sufficient detail to allow replication, including how and when they will be administered<br>L126-1656
	11b	Criteria for discontinuing or modifying allocated interventions for a given trial participant (eg, drug dose change in response to harms, participant request, or improving/worsening disease)<br>L158-169
	11c	Strategies to improve adherence to intervention protocols, and any procedures for monitoring adherence (eg, drug tablet return, laboratory tests)<br>Inapplicable
	11d	Relevant concomitant care and interventions that are permitted or prohibited during the trial

Outcomes	12	Primary, secondary, and other outcomes, including the specific measurement variable (eg, systolic blood pressure), analysis metric (eg, change from baseline, final value, time to event), method of aggregation (eg, median, proportion), and time point for each outcome. Explanation of the clinical relevance of chosen efficacy and harm outcomes is strongly recommended<br>L171-187
Participant timeline	13	Time schedule of enrolment, interventions (including any run-ins and washouts), assessments, and visits for participants. A schematic diagram is highly recommended (see Figure)<br>L174-176
Sample size	14	Estimated number of participants needed to achieve study objectives and how it was determined, including clinical and statistical assumptions supporting any sample size calculations\<br>L189-194
Recruitment	15	Strategies for achieving adequate participant enrolment to reach target sample size<br>L91-93

Methods: Assignment of interventions (for controlled trials)

Allocation:

Sequence generation	16a	Method of generating the allocation sequence (eg, computer-generated random numbers), and list of any factors for stratification. To reduce predictability of a random sequence, details of any planned restriction (eg, blocking) should be provided in a separate document that is unavailable to those who enrol participants or assign interventions<br>L118-124
Allocation concealment mechanism	16b	Mechanism of implementing the allocation sequence (eg, central telephone; sequentially numbered, opaque, sealed envelopes), describing any steps to conceal the sequence until interventions are assigned<br>L118-122
Implementation	16c	Who will generate the allocation sequence, who will enrol participants, and who will assign participants to interventions<br>L112-116
Blinding (masking)	17a	Who will be blinded after assignment to interventions (eg, trial participants, care providers, outcome assessors, data analysts), and how<br>Inapplicable

	17b	If blinded, circumstances under which unblinding is permissible, and procedure for revealing a participant's allocated intervention during the trial<br>Inapplicable

Methods: Data collection, management, and analysis

Data collection methods	18a	Plans for assessment and collection of outcome, baseline, and other trial data, including any related processes to promote data quality (eg, duplicate measurements, training of assessors) and a description of study instruments (eg, questionnaires, laboratory tests) along with their reliability and validity, if known. Reference to where data collection forms can be found, if not in the protocol<br>Please see study protocol<br>http://www.studies-obsgyn.nl/AID-study/page.asp?page_id=1562
	18b	Plans to promote participant retention and complete follow-up, including list of any outcome data to be collected for participants who discontinue or deviate from intervention protocols<br>L161-171
Data management	19	Plans for data entry, coding, security, and storage, including any related processes to promote data quality (eg, double data entry; range checks for data values). Reference to where details of data management procedures can be found, if not in the protocol<br>Please see study protocol<br>http://www.studies-obsgyn.nl/AID-study/page.asp?page_id=1562
Statistical methods	20a	Statistical methods for analysing primary and secondary outcomes. Reference to where other details of the statistical analysis plan can be found, if not in the protocol<br>L198-213
	20b	Methods for any additional analyses (eg, subgroup and adjusted analyses)<br>L198-213
	20c	Definition of analysis population relating to protocol non-adherence (eg, as randomised analysis), and any statistical methods to handle missing data (eg, multiple imputation)<br>L198-213

Methods: Monitoring

Data monitoring	21a	Composition of data monitoring committee (DMC); summary of its role and reporting structure; statement of whether it is independent from the sponsor and competing interests; and reference to where further details about its charter can be found, if not in the protocol. Alternatively, an explanation of why a DMC is not needed<br>Please see study protocol<br>http://www.studies-obsgyn.nl/AID-study/page.asp?page_id=1562

	21b	Description of any interim analyses and stopping guidelines, including who will have access to these interim results and make the final decision to terminate the trial<br>Please see study protocol<br>http://www.studies-obsgyn.nl/AID-study/page.asp?page_id=1562
Harms	22	Plans for collecting, assessing, reporting, and managing solicited and spontaneously reported adverse events and other unintended effects of trial interventions or trial conduct<br>Please see study protocol<br>http://www.studies-obsgyn.nl/AID-study/page.asp?page_id=1562
Auditing	23	Frequency and procedures for auditing trial conduct, if any, and whether the process will be independent from investigators and the sponsor<br>Please see study protocol<br>http://www.studies-obsgyn.nl/AID-study/page.asp?page_id=1562
**Ethics and dissemination**		
Research ethics approval	24	Plans for seeking research ethics committee/institutional review board (REC/IRB) approval<br>L240-244
Protocol amendments	25	Plans for communicating important protocol modifications (eg, changes to eligibility criteria, outcomes, analyses) to relevant parties (eg, investigators, REC/IRBs, trial participants, trial registries, journals, regulators)<br>Please see study protocol<br>http://www.studies-obsgyn.nl/AID-study/page.asp?page_id=1562
Consent or assent	26a	Who will obtain informed consent or assent from potential trial participants or authorised surrogates, and how (see Item 32)<br>L114-118
	26b	Additional consent provisions for collection and use of participant data and biological specimens in ancillary studies, if applicable<br>Inapplicable
Confidentiality	27	How personal information about potential and enrolled participants will be collected, shared, and maintained in order to protect confidentiality before, during, and after the trial<br>Please see study protocol<br>http://www.studies-obsgyn.nl/AID-study/page.asp?page_id=1562
Declaration of interests	28	Financial and other competing interests for principal investigators for the overall trial and each study site<br>L244-246

For peer review only

Access to data	29	Statement of who will have access to the final trial dataset, and disclosure of contractual agreements that limit such access for investigators<br>Please see study protocol<br>http://www.studies-obsgyn.nl/AID-study/page.asp?page_id=1562
Ancillary and post-trial care	30	Provisions, if any, for ancillary and post-trial care, and for compensation to those who suffer harm from trial participation<br>Please see study protocol<br>http://www.studies-obsgyn.nl/AID-study/page.asp?page_id=1562
Dissemination policy	31a	Plans for investigators and sponsor to communicate trial results to participants, healthcare professionals, the public, and other relevant groups (eg, via publication, reporting in results databases, or other data sharing arrangements), including any publication restrictions<br>L244-245
	31b	Authorship eligibility guidelines and any intended use of professional writers<br>Please see study protocol<br>http://www.studies-obsgyn.nl/AID-study/page.asp?page_id=1562
	31c	Plans, if any, for granting public access to the full protocol, participant-level dataset, and statistical code<br>Inapplicable
**Appendices**		
Informed consent materials	32	Model consent form and other related documentation given to participants and authorised surrogates<br>Please see study protocol<br>http://www.studies-obsgyn.nl/AID-study/page.asp?page_id=1562
Biological specimens	33	Plans for collection, laboratory evaluation, and storage of biological specimens for genetic or molecular analysis in the current trial and for future use in ancillary studies, if applicable<br>Inapplicable

*It is strongly recommended that this checklist be read in conjunction with the SPIRIT 2013 Explanation & Elaboration for important clarification on the items. Amendments to the protocol should be tracked and dated. The SPIRIT checklist is copyrighted by the SPIRIT Group under the Creative Commons "Attribution-NonCommercial-NoDerivs 3.0 Unported" license.

For peer review only

BMJ Open

The AID study: protocol for a randomised controlled trial of intrauterine insemination in the natural cycle compared to intracervical insemination in the natural cycle.

Journal:	*BMJ Open*
Manuscript ID	bmjopen-2018-026065.R1
Article Type:	Protocol
Date Submitted by the Author:	22-Mar-2019
Complete List of Authors:	Kop, Petronella; Center for Reproductive Medicine, Amsterdam Reproduction & Development Institute, Amsterdam UMC, University of Amsterdam,<br>van Wely, Madelon; Center for Reproductive Medicine, Amsterdam Reproduction & Development Institute, Amsterdam UMC, University of Amsterdam,<br>Willem Mol, Ben; Monash University Central Clinical School<br>Bernardus, R.E.; Nij Barrahus<br>Brucker, M.; Universitair Ziekenhuis Brussel<br>Janssens, Pim; Hospital Rijnstate, Clinical Chemistry and Haematology<br>Nap, Annemiek; Rijnstate<br>Cohlen, B.J.; Isala Hospitals, Obstetrics and Gynaecology<br>Pieters, Jacqueline; Vivaneo Medisch Centrum Kinderwens<br>Repping, S.; Center for Reproductive Medicine, Amsterdam Reproduction & Development Institute, Amsterdam UMC, University of Amsterdam,<br>van der Veen, F.; Center for Reproductive Medicine, Amsterdam Reproduction & Development Institute, Amsterdam UMC, University of Amsterdam,<br>Mochtar, Monique; Center for Reproductive Medicine, Amsterdam Reproduction & Development Institute, Amsterdam UMC, University of Amsterdam,
**Primary Subject Heading**:	Reproductive medicine
Secondary Subject Heading:	Reproductive medicine
Keywords:	Reproductive medicine < GYNAECOLOGY, intrauterine insemination, intracervical insemination, donor sperm

SCHOLARONE™
Manuscripts

The AID study: protocol for a randomised controlled trial of intrauterine insemination in the natural cycle compared to intracervical insemination in the natural cycle.

Kop Petronella[1], van Wely Madelon[1], Mol Ben[2], Bernardus RE[3], Brucker M. de [4], Janssens Pim[5] , Nap Annemiek[5] , Cohlen BJ[6], Pieters Jacqueline [7], Repping S[1], van der Veen F[1] and Mochtar Monique[1].

[1] Center for Reproductive Medicine, Amsterdam Reproduction & Development Institute, Amsterdam UMC, University of Amsterdam, Amsterdam, The Netherlands
[2] Department of Obstetrics and Gynaecology, Monash University, Melbourne, Australia
[3] Nij Barrahus, Wolvega, The Netherlands
[4] Universitair Ziekenhuis Brussel, Brussels, Belgium
[5] Rijnstate, Arnhem, The Netherlands
[6] Isala Zwolle, Zwolle, The Netherlands
[7] VivaNeo Medisch Centrum Kinderwens, Leiderdorp, The Netherlands

Corresponding author: Ms. Petronella Kop; p.a.kop@amc.uva.nl

ABSTRACT

Introduction At present, studies comparing intrauterine insemination in the natural cycle versus intracervical insemination in the natural cycle in women undergoing artificial insemination with donor sperm are scarce.

Methods and analysis We perform a randomised controlled non-inferiority trial among five secondary and tertiary fertility clinics in the Netherlands and one tertiary fertility clinic in Belgium. Women eligible for artificial insemination with donor sperm are included. We perform six cycles of artificial insemination with donor sperm within a time horizon of eight months comparing intrauterine insemination in the natural cycle with intracervical insemination in the natural cycle. The primary outcome is ongoing pregnancy leading to live birth conceived within eight months after

For peer review only - http://bmjopen.bmj.com/site/about/guidelines.xhtml

randomisation. Secondary outcomes are clinical pregnancy rate, miscarriage rate, multiple pregnancy rate, pregnancy complications (preterm birth, birth weight < 2500 gram, pregnancy induced hypertension, (pre-) eclampsia, HELLP), time to ongoing pregnancy, direct and indirect costs. To demonstrate the non-inferiority of ICI with a margin of 12%, we need 208 women per arm.

Ethics and dissemination The study has been approved by the Medical Ethical Committee of the Academic Medical Centre and from the Dutch Central Committee on Research involving Human Subjects (47330-018-13). Results will be disseminated through peer-reviewed publications and presentations at international scientific meetings.

Trial registration number NTR4462.

Strengths and limitations of this study

- A multicenter randomised controlled design is used
- We aim to give cumulative ongoing pregnancy rates, while previous studies reported clinical pregnancy rates
- Both participants and researchers were not blinded. This can be a limitation.

INTRODUCTION

Artificial insemination with donor sperm (AID) may be performed in heterosexual couples for medical reasons or to assist lesbian couples or single women to achieve pregnancy. Medical reasons for AID include obstructive and non-obstructive azoospermia, severely impaired semen quality in couples who do not wish to undergo or were not successful with ICSI, severe rhesus iso-immunization, prevention of vertical transmission of a genetic defect or prevention of transmission of human immunodeficiency virus (HIV) in couples who do not wish to undergo semen

For peer review only - http://bmjopen.bmj.com/site/about/guidelines.xhtml

washing. [1, 2] Women can be inseminated via the intrauterine (IUI) or the intracervical (ICI) route with or without ovarian stimulation.

A systematic review, in women who started AID, reported intrauterine insemination with ovarian stimulation (IUI-OS) to be more effective than intracervical insemination with ovarian stimulation (ICI-OS) in terms of live birth rate (OR 2.55 95% 0.72-8.96, 1 RCT, n=43) and clinical pregnancy rate (OR 2.83, 95% CI 1.38 to 5.78, 2 RCTs, n=131) at the cost of a higher multiple pregnancy rate (OR 2.77, 95% CI 1.00 to 7.69, 2 RCTs, n=131). In women who started AID in the natural cycle, IUI resulted in similar live birth rates as ICI in the natural cycle (OR 3.24, 95% 0.12-87.13, 1 RCT, n=26) but in higher clinical pregnancy rates in (OR 6.18, 95% CI 1.91-20.03, 2RCTs, n=76). There were no multiple pregnancies. [3]

Multiple pregnancies are associated with increased perinatal and maternal morbidity and perinatal mortality and are regarded as an adverse treatment outcome that is no longer acceptable and should be prevented. [4-6]

Therefore, the single existing guideline on artificial insemination with donor sperm, published by The Royal College of Obstetrics and Gynaecology (RCOG), recommends six cycles of IUI in the natural cycle. [1]

In 2012 we performed a retrospective nationwide cohort study among eight sperm banks in the Netherlands on IUI and ICI in the natural cycle. We included 1843 women of whom 1163 underwent 4269 cycles of IUI in the natural cycle and 680 underwent 2345 cycles of ICI in the natural cycle. Cumulative ongoing pregnancy rates over six treatment cycles were 40.5% for IUI and 37.9% for ICI, resulting in a hazard ratio (HR) of 1.02 (95% CI 0.84-1.23) for IUI versus ICI. [7]

The costs for IUI have been estimated to be four times higher than ICI, mostly because of the sperm processing inherent to IUI. [8]

For peer review only - http://bmjopen.bmj.com/site/about/guidelines.xhtml

Since evidence that underpins the superiority of IUI in the natural cycle over ICI in the natural cycle is scarce and IUI may generate higher costs than ICI for no increase in pregnancies, we propose a multicenter trial comparing IUI and ICI in the natural cycle with a cost effectiveness analysis alongside the trial.

OBJECTIVE

To compare the effectiveness of six cycles of intrauterine insemination in the natural cycle compared with six cycles of intracervical insemination in the natural cycle with cryopreserved donor sperm within a time horizon of eight months.

METHODS AND ANALYSIS

Study design

This study is a non-blinded, multicentre, non-inferiority randomised controlled trial among five secondary and tertiary fertility clinics in the Netherlands and one tertiary fertility clinic in Belgium. Recruitment started on 3 June 2014.

We expect to complete recruitment on 1 January 2020.

Study population

Inclusion criteria

We will study women between 18 and 43 years with a regular cycle, be it spontaneously or after ovulation induction and with an indication for AID. Indications for AID are medical reasons like obstructive and non-obstructive azoospermia, severely impaired semen quality in couples who do not wish to undergo or were not successful with intracytoplasmatic sperm injection (ICSI), severe rhesus iso-immunization, prevention of vertical transmission of a genetic defect or

For peer review only - http://bmjopen.bmj.com/site/about/guidelines.xhtml

For peer review only

prevention of transmission of human immunodeficiency virus in couples who do not wish to undergo semen washing, In addition, lesbian couples or single women who apply for AID are eligible to participate.

Exclusion criteria

Women with known double-sided tubal pathology, irregular menstrual cycles, in vitro fertilisation (IVF) or IUI in their history will not be eligible.

Informed consent procedure

Women eligible for participation in the study are invited for additional counselling by a research nurse to ensure that they are fully informed on the nature of the study by means of both oral and written information. Women who agree to participate are asked to sign a written informed consent of which they receive a copy.

Randomisation

Randomisation is performed by accessing a web-based data system that is used for randomisation in clinical trials and will be performed centrally with the use of a permuted-block-design with randomly selected block sizes that vary between two, four and six.

Couples are randomly allocated to either six cycles of IUI in the natural cycle or six cycles of ICI in the natural cycle.

Interventions

In the IUI cycles, ovulation detection is performed with LH tests or by transvaginal sonography, depending on the local setting. In case of monitoring with urinary LH

For peer review only - http://bmjopen.bmj.com/site/about/guidelines.xhtml

tests, women test their urine once per day, starting on an individually calculated cycle day based on their basal body temperature chart. Women are inseminated with processed semen the day after the endogenous LH surge has been detected in the urine sample. Serum LH measurements are performed from day 11 of the menstrual cycle onwards and women are inseminated one day after the serum LH rise.[9]

In case follicular growth is monitored by transvaginal sonography, timing of insemination can be performed by ovulation triggering or by measuring serum LH levels. In case of ovulation triggering human chorionic gonadotropin (Pregnyl, Organon, Oss, The Netherlands) is administered when the dominant follicle has a diameter of at least 16mm. Women are inseminated 36 to 40 hours thereafter. The semen may be processed in two ways. One way is to process the semen against a density gradient centrifugation and/or a washing step with culture medium according to local laboratory protocol after thawing of the –unprocessed- cryopreserved semen and the second way is to process the semen against a density gradient centrifugation and/or washing step with culture medium according to local laboratory protocol before freezing of the semen.

In the ICI cycles, ovulation detection is in the same way as in the IUI arm and insemination is also timed identical to the IUI arm. Women are inseminated with unprocessed semen once per cycle, and preferably by a cervical cap. [10]

Before starting a new treatment cycle with IUI or ICI, women undergo a pregnancy test (hCG measurement in serum or urine). If the woman is not pregnant the next treatment cycle is started according to protocol. In case of a positive pregnancy test women are monitored by transvaginal sonography. Monitoring will take place at five to nine weeks of amenorrhea to check whether an intrauterine gestational sac is present, i.e. a clinical pregnancy.

For peer review only - http://bmjopen.bmj.com/site/about/guidelines.xhtml

Subsequently, monitoring takes place at 11 to 12 weeks amenorrhea to register the presence of an intrauterine gestational sac with foetal heartbeat, i.e. an ongoing pregnancy.

Follow up

Pregnant women undergo an ultrasound at 7 and 11 weeks of gestation to classify the pregnancy as clinical or ongoing singleton or multiple pregnancy. Women are contacted by telephone to enquire on the course of pregnancy, delivery and the health of the child. Detailed information on maternal complications will be obtained from the responsible obstetrician or midwife. When data on the health of the child are insufficient, child health centres and/or paediatricians are contacted for specific information. Presumably, not all women complete the eight months of treatment. Couples who drop-out will largely represent normal patient flow. In both arms, women will be treated until ongoing pregnancy occurs within a time horizon of eight months. We aim to keep track of all couples who drop-out and to document the reason for the drop-out.

Patient and Public involvement

The Dutch patient organisation for patients with fertility problems (Freya) supports the study. When the study is finished we will request Freya to organise an informal meeting during which fertility experts from our study group will present information on the treatment options. We will ask Freya to publish an easy to read summary of the results on their homepage. The data will thus become available for the public. Study participants are asked if they want to be informed about the results of the study during the informed consent procedure.

For peer review only

Primary outcome measure

The primary outcome is ongoing pregnancy leading to live birth. Ongoing pregnancy is defined as a positive heartbeat at 12 weeks of gestation. Only ongoing pregnancies that occur within the first eight months after randomisation count for assessment of the primary endpoint.

Secondary outcome measures

Secondary outcomes are clinical pregnancy (any registered heartbeat at ultrasound), miscarriage (registered heartbeat before 12 weeks of gestation) and multiple pregnancy (registered heartbeat of at least two foetuses at 12 weeks of gestation) and ectopic pregnancy, time to ongoing pregnancy leading to live birth.
We will register the following pregnancy outcomes: preterm birth < 37 weeks, iatrogenic preterm birth, spontaneous preterm birth, time to delivery, mean birth weight, birth weight < 2.500 gram, growth restriction defined as birthweight < 10th percentile, pregnancy Induced Hypertension (PIH), pre-eclampsia, HELLP-syndrome and direct and indirect costs.

Power calculation

The study is designed as a non-inferiority trial. We assume that six cycles can be completed within a fixed time horizon of eight months. Assuming a live birth rate of 40% after six cycles of IUI, we need 208 women per arm with an alpha of 0.05, and a beta of 0.80 to demonstrate the non-inferiority of ICI with a margin of 12%.[7]
The margin of 12% are the differences shown in literature between IUI and ICI.[3]

Data analysis

For peer review only

We will compare baseline measurements female age, BMI, indication for AID, previous assisted reproductive treatments, previous pregnancies, parity, intoxications and referral status.

The analysis of all outcomes is on an intention-to-treat basis. For ongoing pregnancy we will test non-inferiority on basis of the absolute risk difference with the absolute margin of 12%. We will subsequently estimate differences in the primary and secondary outcomes as relative risks with 95% CI. We will also report p values. We will evaluate association and interaction with our primary outcome of baseline variables in logistic regression analyses. The baseline variables that we plan to include in these analyses are timing, sperm quality and practice variation. We will construct Kaplan-Meier survival curves for time to ongoing pregnancy. For continuous variables, we examine the distribution of the observations, and if normally distributed, we summarize them as means with SDs. If they are not normally distributed, medians and IQRs are reported. For dichotomous data, we provide proportions (or percentages). In addition to the baseline and outcome data, we also summarize the recruitment numbers, those lost to follow-up, protocol violations and other relevant data. We will analyze a maximum of six cycles of IUI or ICI performed within a time horizon of eight months after randomisation.

Data Safety monitoring board

A data safety monitoring board (DSMB) is installed for this trial. In the DSMB professionals from the fields of fertility and epidemiology are represented. All members have independent positions from the trial study group. An interim-analysis

For peer review only - http://bmjopen.bmj.com/site/about/guidelines.xhtml

is performed on the primary endpoint when 200 patients have been randomised and all have completed at least 3 months of follow-up to enable retrieval of the outcome ongoing pregnancy. The interim-analysis will be performed by an independent statistician, blinded for the treatment allocation. The statistician will report to the independent DSMB. The DSMB will decide on the continuation of the trial. There are no safety issues liaised to this trial and futility is welcomed as an outcome. The only reason for premature stopping the trial is a large difference in ongoing pregnancies. We suggest the Peto approach to be used: if an interim analysis shows a probability of less than 0.001 that the treatments are different in terms of ongoing pregnancy, then the trial should be stopped early.

Economic evaluation

We will perform an economic analysis from a healthcare perspective alongside the clinical trial. We make a distinction between direct costs and indirect costs. Direct costs include the costs of medication, cycle monitoring, interventions and the costs of pregnancy leading to live birth. Indirect costs are collected from the individual case report forms of the RCT. For each woman, we registered cycle monitoring (number of ultrasounds), we registered the medication, and interventions (cycles with IUI, cycles with ICI) they received within six subsequent cycles or until an ongoing pregnancy occurred within a time horizon of eight months.

Societal costs of travel and time will be determined on basis of recourse use as registered in the case report form.

We will analyse a cost-minimization or cost-effectiveness analysis depending on the outcome of ongoing pregnancy rates in both groups. We present the cost-effectiveness of each strategy as cost per ongoing pregnancy and costs per live birth.

For peer review only

We explore the robustness of the results for various assumptions and parameter estimates in sensitivity analysis outcomes and we express these in incremental cost-effectiveness ratio graphs and cost-effectiveness acceptability curves. The economic evaluation will be reported in a separate paper.

DISCUSSION

In women who start AID, there is uncertainty about the most cost effective treatment modality. IUI-OS leads to higher clinical pregnancy rates compared to ICI-OS, but the incidence of multiple pregnancies with the addition of OS is substantial and hardly acceptable.[3] Studies on IUI and ICI in the natural cycle report no differences in live birth rate between IUI and ICI in natural cycles, but higher clinical pregnancy rates for IUI, without any multiple pregnancies. Therefore, the NICE guideline recommends IUI in the natural cycle for the first six cycles. [1] Since IUI compared to ICI in natural cycles shows no difference in live birth rate, IUI may generate higher costs than ICI for no increase in live birth rates. There are no large randomised controlled trials that addresses this issue by comparing IUI in the natural cycle with ICI in the natural cycle in women who start with AID.

ETHICS AND DISSEMINATION

The study has been approved by the Medical Ethical Committee of the Academic Medical Centre and from the Dutch Central Committee on Research involving Human Subjects (47330-018-13). The boards of the participating hospitals approved the study. Results will be disseminated through peer-reviewed publications and presentations at international scientific meeting

For peer review only - http://bmjopen.bmj.com/site/about/guidelines.xhtml

References

1. *National institute for Health and Care Excellence (NICE). Fertility: Assessment and treatment for people with fertility problems*. 2013.
2. Practice Committee of American Society for Reproductive, M. and T. Practice Committee of Society for Assisted Reproductive, *Recommendations for gamete and embryo donation: a committee opinion.* Fertil Steril, 2013. **99**(1): p. 47-62.
3. Kop, P.A., et al., *Intrauterine insemination versus intracervical insemination in donor sperm treatment.* Cochrane Database Syst Rev, 2018. **1**: p. CD000317.
4. Cohlen, B., et al., *Multiple pregnancy after assisted reproduction.* Lancet, 2005. **366**(9484): p. 452-3; author reply 453-4.
5. Fauser, B.C., P. Devroey, and N.S. Macklon, *Multiple birth resulting from ovarian stimulation for subfertility treatment.* Lancet, 2005. **365**(9473): p. 1807-16.
6. Nyboe Andersen, A., et al., *Assisted reproductive technology in Europe, 2000. Results generated from European registers by ESHRE.* Hum Reprod, 2004. **19**(3): p. 490-503.
7. Kop, P.A., et al., *Intrauterine insemination or intracervical insemination with cryopreserved donor sperm in the natural cycle: a cohort study.* Human Reproduction, 2015. **30**(3): p. 603-7.
8. *Nederlandse zorgautoriteit*. 2012; http://www.nza.nl/zorgonderwerpen/zorgonderwerpen/ziekenhuiszorg/veelgesteldevragen/veelgestelde-vragen-dbc-dot/, .
9. Blockeel, C., et al., *Should an intrauterine insemination with donor semen be performed 1 or 2 days after the spontaneous LH rise? A prospective RCT.* Human Reproduction, 2014. **29**(4): p. 697-703.
10. Flierman PA, Hogerzeil HV, Hemrika DJ., A prospective, randomised, cross-over comparison of two methods of artificial insemination by donor on the incidence of conception: intracervical insemination by straw versus cervical cap. Human reproduction, 1997. 12 (9): p. 1945-8.

Funding statement

This trial will receive funding from the Netherlands Organization for Health Research and Development (ZonMw project number 837002407)). There is no role in the study for ZonMw.

Author’s contributions

PALK is responsible for the overall logistical aspects of the trial and drafted the paper. MvW, MHM, FvdV, SR and BWM designed the trial and were responsible for the development of the protocol. PALK, REB, MB, PMWJ, AN, BJC, JJPMP, MvW, MHM, SR, BMW and FvdV contributed to the protocol included patients and approved the final version of the paper.

For peer review only - http://bmjopen.bmj.com/site/about/guidelines.xhtml

Competing interest: None declared

For peer review only

SPIRIT 2013 Checklist: Recommended items to address in a clinical trial protocol and related documents*

Section/item	Item No	Description
**Administrative information**		
Title	1	Descriptive title identifying the study design, population, interventions, and, if applicable, trial acronym<br>L2-4
Trial registration	2a	Trial identifier and registry name. If not yet registered, name of intended registry<br>L41
	2b	All items from the World Health Organization Trial Registration Data Set<br>L41
Protocol version	3	Date and version identifier<br>Footnote
Funding	4	Sources and types of financial, material, and other support<br>L273-277
Roles and responsibilities	5a	Names, affiliations, and roles of protocol contributors<br>Footnotes L248-252
	5b	Name and contact information for the trial sponsor<br>L278-281
	5c	Role of study sponsor and funders, if any, in study design; collection, management, analysis, and interpretation of data; writing of the report; and the decision to submit the report for publication, including whether they will have ultimate authority over any of these activities<br>L278-281
	5d	Composition, roles, and responsibilities of the coordinating centre, steering committee, endpoint adjudication committee, data management team, and other individuals or groups overseeing the trial, if applicable (see Item 21a for data monitoring committee)<br>L94-97
**Introduction**		

For peer review only - http://bmjopen.bmj.com/site/about/guidelines.xhtml

Background and rationale	6a	Description of research question and justification for undertaking the trial, including summary of relevant studies (published and unpublished) examining benefits and harms for each intervention<br>L50-90
	6b	Explanation for choice of comparators<br>L82-85
Objectives	7	Specific objectives or hypotheses<br>L88-90
Trial design	8	Description of trial design including type of trial (eg, parallel group, crossover, factorial, single group), allocation ratio, and framework (eg, superiority, equivalence, noninferiority, exploratory)<br>L94-97
**Methods: Participants, interventions, and outcomes**		
Study setting	9	Description of study settings (eg, community clinic, academic hospital) and list of countries where data will be collected. Reference to where list of study sites can be obtained<br>L94-96
Eligibility criteria	10	Inclusion and exclusion criteria for participants. If applicable, eligibility criteria for study centres and individuals who will perform the interventions (eg, surgeons, psychotherapists)<br>L101-113
Interventions	11a	Interventions for each group with sufficient detail to allow replication, including how and when they will be administered<br>L122-159
	11b	Criteria for discontinuing or modifying allocated interventions for a given trial participant (eg, drug dose change in response to harms, participant request, or improving/worsening disease)<br>L162-172
	11c	Strategies to improve adherence to intervention protocols, and any procedures for monitoring adherence (eg, drug tablet return, laboratory tests)<br>Inapplicable
	11d	Relevant concomitant care and interventions that are permitted or prohibited during the trial

For peer review only - http://bmjopen.bmj.com/site/about/guidelines.xhtml

Outcomes	12	Primary, secondary, and other outcomes, including the specific measurement variable (eg, systolic blood pressure), analysis metric (eg, change from baseline, final value, time to event), method of aggregation (eg, median, proportion), and time point for each outcome. Explanation of the clinical relevance of chosen efficacy and harm outcomes is strongly recommended<br>L182-196
Participant timeline	13	Time schedule of enrolment, interventions (including any run-ins and washouts), assessments, and visits for participants. A schematic diagram is highly recommended (see Figure)<br>L182-183
Sample size	14	Estimated number of participants needed to achieve study objectives and how it was determined, including clinical and statistical assumptions supporting any sample size calculations\<br>L199-203
Recruitment	15	Strategies for achieving adequate participant enrolment to reach target sample size<br>L94-97

Methods: Assignment of interventions (for controlled trials)

Allocation:

Sequence generation	16a	Method of generating the allocation sequence (eg, computer-generated random numbers), and list of any factors for stratification. To reduce predictability of a random sequence, details of any planned restriction (eg, blocking) should be provided in a separate document that is unavailable to those who enrol participants or assign interventions<br>L118-124
Allocation concealment mechanism	16b	Mechanism of implementing the allocation sequence (eg, central telephone; sequentially numbered, opaque, sealed envelopes), describing any steps to conceal the sequence until interventions are assigned<br>L122-127
Implementation	16c	Who will generate the allocation sequence, who will enrol participants, and who will assign participants to interventions<br>L116-119
Blinding (masking)	17a	Who will be blinded after assignment to interventions (eg, trial participants, care providers, outcome assessors, data analysts), and how<br>Inapplicable

	17b	If blinded, circumstances under which unblinding is permissible, and procedure for revealing a participant's allocated intervention during the trial<br>Inapplicable

Methods: Data collection, management, and analysis

Data collection methods	18a	Plans for assessment and collection of outcome, baseline, and other trial data, including any related processes to promote data quality (eg, duplicate measurements, training of assessors) and a description of study instruments (eg, questionnaires, laboratory tests) along with their reliability and validity, if known. Reference to where data collection forms can be found, if not in the protocol<br>Please see study protocol<br>http://www.studies-obsgyn.nl/AID-study/page.asp?page_id=1562
	18b	Plans to promote participant retention and complete follow-up, including list of any outcome data to be collected for participants who discontinue or deviate from intervention protocols<br>L162-172
Data management	19	Plans for data entry, coding, security, and storage, including any related processes to promote data quality (eg, double data entry; range checks for data values). Reference to where details of data management procedures can be found, if not in the protocol<br>Please see study protocol<br>http://www.studies-obsgyn.nl/AID-study/page.asp?page_id=1562
Statistical methods	20a	Statistical methods for analysing primary and secondary outcomes. Reference to where other details of the statistical analysis plan can be found, if not in the protocol<br>L206-223
	20b	Methods for any additional analyses (eg, subgroup and adjusted analyses)<br>L213-214
	20c	Definition of analysis population relating to protocol non-adherence (eg, as randomised analysis), and any statistical methods to handle missing data (eg, multiple imputation)<br>L220-222

Methods: Monitoring

Data monitoring	21a	Composition of data monitoring committee (DMC); summary of its role and reporting structure; statement of whether it is independent from the sponsor and competing interests; and reference to where further details about its charter can be found, if not in the protocol. Alternatively, an explanation of why a DMC is not needed<br>Line 225-238

	21b	Description of any interim analyses and stopping guidelines, including who will have access to these interim results and make the final decision to terminate the trial<br>Please see study protocol<br>http://www.studies-obsgyn.nl/AID-study/page.asp?page_id=1562
Harms	22	Plans for collecting, assessing, reporting, and managing solicited and spontaneously reported adverse events and other unintended effects of trial interventions or trial conduct<br>Please see study protocol<br>http://www.studies-obsgyn.nl/AID-study/page.asp?page_id=1562
Auditing	23	Frequency and procedures for auditing trial conduct, if any, and whether the process will be independent from investigators and the sponsor<br>Please see study protocol<br>http://www.studies-obsgyn.nl/AID-study/page.asp?page_id=1562
**Ethics and dissemination**		
Research ethics approval	24	Plans for seeking research ethics committee/institutional review board (REC/IRB) approval<br>L273-277
Protocol amendments	25	Plans for communicating important protocol modifications (eg, changes to eligibility criteria, outcomes, analyses) to relevant parties (eg, investigators, REC/IRBs, trial participants, trial registries, journals, regulators)<br>Please see study protocol<br>http://www.studies-obsgyn.nl/AID-study/page.asp?page_id=1562
Consent or assent	26a	Who will obtain informed consent or assent from potential trial participants or authorised surrogates, and how (see Item 32)<br>L116-
	26b	Additional consent provisions for collection and use of participant data and biological specimens in ancillary studies, if applicable<br>Inapplicable
Confidentiality	27	How personal information about potential and enrolled participants will be collected, shared, and maintained in order to protect confidentiality before, during, and after the trial<br>Please see study protocol<br>http://www.studies-obsgyn.nl/AID-study/page.asp?page_id=1562
Declaration of interests	28	Financial and other competing interests for principal investigators for the overall trial and each study site<br>L244-246

For peer review only - http://bmjopen.bmj.com/site/about/guidelines.xhtml

Access to data	29	Statement of who will have access to the final trial dataset, and disclosure of contractual agreements that limit such access for investigators<br>Please see study protocol<br>http://www.studies-obsgyn.nl/AID-study/page.asp?page_id=1562
Ancillary and post-trial care	30	Provisions, if any, for ancillary and post-trial care, and for compensation to those who suffer harm from trial participation<br>Please see study protocol<br>http://www.studies-obsgyn.nl/AID-study/page.asp?page_id=1562
Dissemination policy	31a	Plans for investigators and sponsor to communicate trial results to participants, healthcare professionals, the public, and other relevant groups (eg, via publication, reporting in results databases, or other data sharing arrangements), including any publication restrictions<br>L174-180
	31b	Authorship eligibility guidelines and any intended use of professional writers<br>Please see study protocol<br>http://www.studies-obsgyn.nl/AID-study/page.asp?page_id=1562
	31c	Plans, if any, for granting public access to the full protocol, participant-level dataset, and statistical code<br>Inapplicable
**Appendices**		
Informed consent materials	32	Model consent form and other related documentation given to participants and authorised surrogates<br>Please see study protocol<br>http://www.studies-obsgyn.nl/AID-study/page.asp?page_id=1562
Biological specimens	33	Plans for collection, laboratory evaluation, and storage of biological specimens for genetic or molecular analysis in the current trial and for future use in ancillary studies, if applicable<br>Inapplicable

*It is strongly recommended that this checklist be read in conjunction with the SPIRIT 2013 Explanation & Elaboration for important clarification on the items. Amendments to the protocol should be tracked and dated. The SPIRIT checklist is copyrighted by the SPIRIT Group under the Creative Commons "Attribution-NonCommercial-NoDerivs 3.0 Unported" license.

For peer review only

BMJ Open

The AID study: protocol for a randomised controlled trial of intrauterine insemination in the natural cycle compared to intracervical insemination in the natural cycle.

Journal:	*BMJ Open*
Manuscript ID	bmjopen-2018-026065.R2
Article Type:	Protocol
Date Submitted by the Author:	04-Jul-2019

Complete List of Authors:	Kop, Petronella; Center for Reproductive Medicine, Amsterdam Reproduction & Development Institute, Amsterdam UMC, University of Amsterdam,<br>van Wely, Madelon; Center for Reproductive Medicine, Amsterdam Reproduction & Development Institute, Amsterdam UMC, University of Amsterdam,<br>Nap, Annemiek; Rijnstate<br>Willem Mol, Ben; Monash University Central Clinical School<br>Bernardus, R.E.; Nij Barrahus<br>Brucker, M.; Universitair Ziekenhuis Brussel<br>Janssens, Pim; Hospital Rijnstate, Clinical Chemistry and Haematology<br>Cohlen, B.J.; Isala Hospitals, Obstetrics and Gynaecology<br>Pieters, Jacqueline; Vivaneo Medisch Centrum Kinderwens<br>Repping, S.; Center for Reproductive Medicine, Amsterdam Reproduction & Development Institute, Amsterdam UMC, University of Amsterdam,<br>van der Veen, F.; Center for Reproductive Medicine, Amsterdam Reproduction & Development Institute, Amsterdam UMC, University of Amsterdam,<br>Mochtar, Monique; Center for Reproductive Medicine, Amsterdam Reproduction & Development Institute, Amsterdam UMC, University of Amsterdam,

**Primary Subject Heading**:	Reproductive medicine
Secondary Subject Heading:	Reproductive medicine
Keywords:	Reproductive medicine < GYNAECOLOGY, intrauterine insemination, intracervical insemination, donor sperm

SCHOLARONE™
Manuscripts

Manuscript protocol AID study BMJ Open June 2019 version 4.0

The AID study: protocol for a randomised controlled trial of intrauterine insemination in the natural cycle compared to intracervical insemination in the natural cycle.

PAL Kop[1], M van Wely[1], A Nap[2], BW Mol[3], RE Bernardus[4], M De Brucker[5], PMW Janssens[2], BJ Cohlen[7], J.J.P.M. Pieters [8], S Repping[1], F van der Veen[1] and MH Mochtar[1].

[1] Center for Reproductive Medicine, Amsterdam Reproduction & Development Institute, Amsterdam UMC, University of Amsterdam, Amsterdam, The Netherlands

[2] Rijnstate, Arnhem, The Netherlands

[3] Department of Obstetrics and Gynaecology, Monash University, Melbourne, Australia

[4] Nij Barrahus, Wolvega, The Netherlands

[5] Universitair Ziekenhuis Brussel, Brussels, Belgium

[6] Rijnstate, Arnhem, The Netherlands

[7] Isala Zwolle, Zwolle, The Netherlands

[8] VivaNeo Medisch Centrum Kinderwens, Leiderdorp, The Netherlands

Corresponding author: P.A.L. Kop, p.a.kop@amc.uva.nl

ABSTRACT

Introduction At present, studies comparing intrauterine insemination in the natural cycle versus intracervical insemination in the natural cycle in women undergoing artificial insemination with donor sperm are scarce.

Methods and analysis We perform a randomised controlled non-inferiority trial among five secondary and tertiary fertility clinics in the Netherlands and one tertiary fertility clinic in Belgium. Women eligible for artificial insemination with donor sperm are included. We perform six cycles of artificial insemination with donor sperm within a time horizon of eight months comparing intrauterine insemination in the natural cycle with intracervical insemination in the natural cycle. The primary outcome is

For peer review only - http://bmjopen.bmj.com/site/about/guidelines.xhtml

ongoing pregnancy leading to live birth conceived within eight months after randomisation. Secondary outcomes are clinical pregnancy rate, miscarriage rate, multiple pregnancy rate, pregnancy complications (preterm birth, birth weight < 2500 gram, pregnancy induced hypertension, (pre-) eclampsia, HELLP), time to ongoing pregnancy, direct and indirect costs. To demonstrate the non-inferiority of ICI with a margin of 12%, we need 208 women per arm.

Ethics and dissemination The study has been approved by the Medical Ethical Committee of the Academic Medical Centre and from the Dutch Central Committee on Research involving Human Subjects (47330-018-13). Results will be disseminated through peer-reviewed publications and presentations at international scientific meetings.

Trial registration number NTR4462.

Strengths and limitations of this study

- A multicenter randomised controlled design is used
- We aim to give cumulative ongoing pregnancy rates, while previous studies reported clinical pregnancy rates
- A limitation is that participants and researchers were not blinded.

INTRODUCTION

Artificial insemination with donor sperm (AID) may be performed in heterosexual couples for medical reasons or to assist lesbian couples or single women to achieve pregnancy. Medical reasons for AID include obstructive and non-obstructive azoospermia, severely impaired semen quality in couples who do not wish to undergo or were not successful with ICSI, severe rhesus iso-immunization, prevention of vertical transmission of a genetic defect or prevention of transmission of human immunodeficiency virus (HIV) in couples who do not wish to undergo semen

For peer review only - http://bmjopen.bmj.com/site/about/guidelines.xhtml

washing. [1, 2] Women can be inseminated via the intrauterine (IUI) or the intracervical (ICI) route with or without ovarian stimulation.

A systematic review, in women who started AID, reported intrauterine insemination with ovarian stimulation (IUI-OS) to be more effective than intracervical insemination with ovarian stimulation (ICI-OS) in terms of live birth rate (OR 2.55 95% 0.72-8.96, 1 RCT, n=43) and clinical pregnancy rate (OR 2.83, 95% CI 1.38 to 5.78, 2 RCTs, n=131) at the cost of a higher multiple pregnancy rate (OR 2.77, 95% CI 1.00 to 7.69, 2 RCTs, n=131). In women who started AID in the natural cycle, IUI resulted in similar live birth rates as ICI in the natural cycle (OR 3.24, 95% 0.12-87.13, 1 RCT, n=26) but in higher clinical pregnancy rates in (OR 6.18, 95% CI 1.91-20.03, 2RCTs, n=76). There were no multiple pregnancies. [3]

Multiple pregnancies are associated with increased perinatal and maternal morbidity and perinatal mortality and are regarded as an adverse treatment outcome that is no longer acceptable and should be prevented. [4-6]

Therefore, the single existing guideline on artificial insemination with donor sperm, published by The Royal College of Obstetrics and Gynaecology (RCOG), recommends six cycles of IUI in the natural cycle. [1]

In 2012 we performed a retrospective nationwide cohort study among eight sperm banks in the Netherlands on IUI and ICI in the natural cycle. We included 1843 women of whom 1163 underwent 4269 cycles of IUI in the natural cycle and 680 underwent 2345 cycles of ICI in the natural cycle. Cumulative ongoing pregnancy rates over six treatment cycles were 40.5% for IUI and 37.9% for ICI, resulting in a hazard ratio (HR) of 1.02 (95% CI 0.84-1.23) for IUI versus ICI. [7]

The costs for IUI have been estimated to be four times higher than ICI, mostly because of the sperm processing inherent to IUI. [8]

For peer review only - http://bmjopen.bmj.com/site/about/guidelines.xhtml

Since evidence that underpins the superiority of IUI in the natural cycle over ICI in the natural cycle is scarce and IUI may generate higher costs than ICI for no increase in pregnancies, we propose a multicenter trial comparing IUI and ICI in the natural cycle with a cost effectiveness analysis alongside the trial.

OBJECTIVE

To compare the effectiveness of six cycles of intrauterine insemination in the natural cycle compared with six cycles of intracervical insemination in the natural cycle with cryopreserved donor sperm within a time horizon of eight months.

METHODS AND ANALYSIS

Study design

This study is a non-blinded, multicentre, non-inferiority randomised controlled trial among five secondary and tertiary fertility clinics in the Netherlands and one tertiary fertility clinic in Belgium. Recruitment started on 3 June 2014.

We expect to complete recruitment on 1 January 2020.

Study population

Inclusion criteria

We will study women between 18 and 43 years with a regular cycle, be it spontaneously or after ovulation induction and with an indication for AID.

Indications for AID are medical reasons like obstructive and non-obstructive azoospermia, severely impaired semen quality in couples who do not wish to undergo or were not successful with intracytoplasmatic sperm injection (ICSI), severe rhesus iso-immunization, prevention of vertical transmission of a genetic defect or

For peer review only

For peer review only - http://bmjopen.bmj.com/site/about/guidelines.xhtml

prevention of transmission of human immunodeficiency virus in couples who do not wish to undergo semen washing, In addition, lesbian couples or single women who apply for AID are eligible to participate.

Exclusion criteria

Women with known double-sided tubal pathology, irregular menstrual cycles, in vitro fertilisation (IVF) or IUI in their history will not be eligible.

Informed consent procedure

Women eligible for participation in the study are invited for additional counselling by a research nurse to ensure that they are fully informed on the nature of the study by means of both oral and written information. Women who agree to participate are asked to sign a written informed consent of which they receive a copy.

Randomisation

Randomisation is performed by accessing a web-based data system that is used for randomisation in clinical trials and will be performed centrally with the use of a permuted-block-design with randomly selected block sizes that vary between two, four and six. We used a permuted block design to randomly allocate participants to a treatment and aimed to maintain balance across treatment groups. In this way, the clinician who performs the randomisation cannot predict the next randomisation to follow. Patients are randomised individually. Patients are randomly allocated to either six cycles of IUI in the natural cycle or six cycles of ICI in the natural cycle.

Interventions

For peer review only - http://bmjopen.bmj.com/site/about/guidelines.xhtml

In the IUI cycles, ovulation detection is performed with LH tests or by transvaginal sonography, depending on the local setting. In case of monitoring with urinary LH tests, women test their urine once per day, starting on an individually calculated cycle day based on their basal body temperature chart. Women are inseminated with processed semen the day after the endogenous LH surge has been detected in the urine sample. Serum LH measurements are performed from day 11 of the menstrual cycle onwards and women are inseminated one day after the serum LH rise.[9]

In case follicular growth is monitored by transvaginal sonography, timing of insemination can be performed by ovulation triggering or by measuring serum LH levels. In case of ovulation triggering human chorionic gonadotropin (Pregnyl, Organon, Oss, The Netherlands) is administered when the dominant follicle has a diameter of at least 16mm. Women are inseminated 36 to 40 hours thereafter. The semen may be processed in two ways. One way is to process the semen against a density gradient centrifugation and/or a washing step with culture medium according to local laboratory protocol after thawing of the –unprocessed- cryopreserved semen and the second way is to process the semen against a density gradient centrifugation and/or washing step with culture medium according to local laboratory protocol before freezing of the semen.

In the ICI cycles, ovulation detection is in the same way as in the IUI arm and insemination is also timed identical to the IUI arm. Women are inseminated with unprocessed semen once per cycle, and preferably by a cervical cap. [6]

Before starting a new treatment cycle with IUI or ICI, women undergo a pregnancy test (hCG measurement in serum or urine). If the woman is not pregnant the next treatment cycle is started according to protocol. In case of a positive pregnancy test women are monitored by transvaginal sonography. Monitoring will take place at five

For peer review only

For peer review only - http://bmjopen.bmj.com/site/about/guidelines.xhtml

to nine weeks of amenorrhea to check whether an intrauterine gestational sac is present, i.e. a clinical pregnancy.

Subsequently, monitoring takes place at 11 to 12 weeks amenorrhea to register the presence of an intrauterine gestational sac with foetal heartbeat, i.e. an ongoing pregnancy.

Follow up

Pregnant women undergo an ultrasound at 7 and 11 weeks of gestation to classify the pregnancy as clinical or ongoing singleton or multiple pregnancy. Women are contacted by telephone to enquire on the course of pregnancy, delivery and the health of the child. Detailed information on maternal complications will be obtained from the responsible obstetrician or midwife. When data on the health of the child are insufficient, child health centres and/or paediatricians are contacted for specific information. Presumably, not all women complete the eight months of treatment. Couples who drop-out will largely represent normal patient flow. In both arms, women will be treated until ongoing pregnancy occurs within a time horizon of eight months. We aim to keep track of all couples who drop-out and to document the reason for the drop-out.

Patient and Public involvement

The Dutch patient organisation for patients with fertility problems (Freya) supports the study. When the study is finished we will request Freya to organise an informal meeting during which fertility experts from our study group will present information on the treatment options. We will ask Freya to publish an easy to read summary of the results on their homepage. The data will thus become available for the public. Study

For peer review only

participants are asked if they want to be informed about the results of the study during the informed consent procedure.

Primary outcome measure

The primary outcome is ongoing pregnancy leading to live birth. Ongoing pregnancy is defined as a positive heartbeat at 12 weeks of gestation. Only ongoing pregnancies that occur within the first eight months after randomisation count for assessment of the primary endpoint.

Secondary outcome measures

Secondary outcomes are clinical pregnancy (any registered heartbeat at ultrasound), miscarriage (registered heartbeat before 12 weeks of gestation) and multiple pregnancy (registered heartbeat of at least two foetuses at 12 weeks of gestation) and ectopic pregnancy, time to ongoing pregnancy leading to live birth.

We will register the following pregnancy outcomes: preterm birth < 37 weeks, iatrogenic preterm birth, spontaneous preterm birth, time to delivery, mean birth weight, birth weight < 2.500 gram, growth restriction defined as birthweight < 10th percentile, pregnancy Induced Hypertension (PIH), pre-eclampsia, HELLP-syndrome and direct and indirect costs.

Power calculation

The study is designed as a non-inferiority trial. We assume that six cycles can be completed within a fixed time horizon of eight months. Assuming a live birth rate of 40% after six cycles of IUI, we need 208 women per arm with an alpha of 0.05, and a beta of 0.80 to demonstrate the non-inferiority of ICI with a margin of 12%.[7]

For peer review only - http://bmjopen.bmj.com/site/about/guidelines.xhtml

The margin of 12% are the differences shown in literature between IUI and ICI. [3]

A limitation of our study is that we use a non-inferiority margin of 12%. Most clinicians and patients would feel that 12% is a rather large and clinically relevant difference.

Data analysis

We will compare baseline measurements female age, BMI, indication for AID, previous assisted reproductive treatments, previous pregnancies, parity, intoxications and referral status.

The analysis of all outcomes is on an intention-to-treat basis. For ongoing pregnancy we will test non-inferiority on basis of the absolute risk difference with the absolute margin of 12%. We will subsequently estimate differences in the primary and secondary outcomes as relative risks with 95% CI. We will also report p values. We will evaluate association and interaction with our primary outcome of baseline variables in logistic regression analyses. The baseline variables that we plan to include in these analyses are timing, sperm quality and practice variation. We will construct Kaplan-Meier survival curves for time to ongoing pregnancy. For continuous variables, we examine the distribution of the observations, and if normally distributed, we summarize them as means with SDs. If they are not normally distributed, medians and IQRs are reported. For dichotomous data, we provide proportions (or percentages). In addition to the baseline and outcome data, we also summarize the recruitment numbers, those lost to follow-up, protocol violations and other relevant data. We will analyze a maximum of six cycles of IUI or ICI performed within a time horizon of eight months after randomisation.

For peer review only - http://bmjopen.bmj.com/site/about/guidelines.xhtml

For peer review only

Data Safety monitoring board

A data safety monitoring board (DSMB) is installed for this trial. In the DSMB professionals from the fields of fertility and epidemiology are represented. All members have independent positions from the trial study group. An interim-analysis is performed on the primary endpoint when 200 patients have been randomised and all have completed at least 3 months of follow-up to enable retrieval of the outcome ongoing pregnancy. The interim-analysis will be performed by an independent statistician, blinded for the treatment allocation. The statistician will report to the independent DSMB. The DSMB will decide on the continuation of the trial. There are no safety issues liaised to this trial and futility is welcomed as an outcome. The only reason for premature stopping the trial is a large difference in ongoing pregnancies. We suggest the Peto approach to be used: if an interim analysis shows a probability of less than 0.001 that the treatments are different in terms of ongoing pregnancy, then the trial should be stopped early.

Economic evaluation

We will perform an economic analysis from a healthcare perspective alongside the clinical trial. We make a distinction between direct costs and indirect costs. Direct costs include the costs of medication, cycle monitoring, interventions and the costs of pregnancy leading to live birth. Indirect costs are collected from the individual case report forms of the RCT. For each woman, we registered cycle monitoring (number of ultrasounds), we registered the medication, and interventions (cycles with IUI, cycles with ICI) they received within six subsequent cycles or until an ongoing pregnancy occurred within a time horizon of eight months.

For peer review only - http://bmjopen.bmj.com/site/about/guidelines.xhtml

Societal costs of travel and time will be determined on basis of recourse use as registered in the case report form.

We will analyse a cost-minimization or cost-effectiveness analysis depending on the outcome of ongoing pregnancy rates in both groups. We present the cost-effectiveness of each strategy as cost per ongoing pregnancy and costs per live birth. We explore the robustness of the results for various assumptions and parameter estimates in sensitivity analysis outcomes and we express these in incremental cost-effectiveness ratio graphs and cost-effectiveness acceptability curves. The economic evaluation will be reported in a separate paper.

DISCUSSION

In women who start AID, there is uncertainty about the most cost effective treatment modality. IUI-OS leads to higher clinical pregnancy rates compared to ICI-OS, but the incidence of multiple pregnancies with the addition of OS is substantial and hardly acceptable.[3] Studies on IUI and ICI in the natural cycle report no differences in live birth rate between IUI and ICI in natural cycles, but higher clinical pregnancy rates for IUI, without any multiple pregnancies. Therefore, the NICE guideline recommends IUI in the natural cycle for the first six cycles. [1] Since IUI compared to ICI in natural cycles shows no difference in live birth rate, IUI may generate higher costs than ICI for no increase in live birth rates. There are no large randomised controlled trials that addresses this issue by comparing IUI in the natural cycle with ICI in the natural cycle in women who start with AID.

ETHICS AND DISSEMINATION

For peer review only - http://bmjopen.bmj.com/site/about/guidelines.xhtml

The study has been approved by the Medical Ethical Committee of the Academic Medical Centre and from the Dutch Central Committee on Research involving Human Subjects (47330-018-13). The boards of the participating hospitals approved the study. Results will be disseminated through peer-reviewed publications and presentations at international scientific meeting

For peer review only

For peer review only - http://bmjopen.bmj.com/site/about/guidelines.xhtml

References

1. *National institute for Health and Care Excellence (NICE). Fertility: Assessment and treatment for people with fertility problems*. 2013.
2. Practice Committee of American Society for Reproductive, M. and T. Practice Committee of Society for Assisted Reproductive, *Recommendations for gamete and embryo donation: a committee opinion.* Fertil Steril, 2013. **99**(1): p. 47-62.
3. Kop, P.A., et al., *Intrauterine insemination versus intracervical insemination in donor sperm treatment.* Cochrane Database Syst Rev, 2018. **1**: p. CD000317.
4. Cohlen, B., et al., *Multiple pregnancy after assisted reproduction.* Lancet, 2005. **366**(9484): p. 452-3; author reply 453-4.
5. Fauser, B.C., P. Devroey, and N.S. Macklon, *Multiple birth resulting from ovarian stimulation for subfertility treatment.* Lancet, 2005. **365**(9473): p. 1807-16.
6. Nyboe Andersen, A., et al., *Assisted reproductive technology in Europe, 2000. Results generated from European registers by ESHRE.* Hum Reprod, 2004. **19**(3): p. 490-503.
7. Kop, P.A., et al., *Intrauterine insemination or intracervical insemination with cryopreserved donor sperm in the natural cycle: a cohort study.* Human Reproduction, 2015. **30**(3): p. 603-7.
8. *Nederlandse zorgautoriteit*. 2012; http://www.nza.nl/zorgonderwerpen/zorgonderwerpen/ziekenhuiszorg/veelgesteldevragen/veelgestelde-vragen-dbc-dot/,].
9. Blockeel, C., et al., *Should an intrauterine insemination with donor semen be performed 1 or 2 days after the spontaneous LH rise? A prospective RCT.* Human Reproduction, 2014. **29**(4): p. 697-703.

Funding statement

This trial will receive funding from the Netherlands Organization for Health Research and Development (ZonMw project number 837002407)). There is no role in the study for ZonMw.

Author’s contributions

PALK is responsible for the overall logistical aspects of the trial and drafted the paper. MvW, MHM, FvdV, SR and BWM designed the trial and were responsible for the development of the protocol. PALK, REB, MB, PMWJ, AN, BJC, JJPMP, MvW, MHM, SR, BMW and FvdV contributed to the protocol included patients and approved the final version of the paper.

For peer review only - http://bmjopen.bmj.com/site/about/guidelines.xhtml

Competing interest: None declared

For peer review only

For peer review only - http://bmjopen.bmj.com/site/about/guidelines.xhtml

SPIRIT

STANDARD PROTOCOL ITEMS: RECOMMENDATIONS FOR INTERVENTIONAL TRIALS

SPIRIT 2013 Checklist: Recommended items to address in a clinical trial protocol and related documents*

Section/item	Item No	Description
**Administrative information**		
Title	1	Descriptive title identifying the study design, population, interventions, and, if applicable, trial acronym<br>L2-4
Trial registration	2a	Trial identifier and registry name. If not yet registered, name of intended registry<br>L41
	2b	All items from the World Health Organization Trial Registration Data Set<br>L41
Protocol version	3	Date and version identifier<br>Footnote
Funding	4	Sources and types of financial, material, and other support<br>L283-285
Roles and responsibilities	5a	Names, affiliations, and roles of protocol contributors<br>Footnotes L287-300
	5b	Name and contact information for the trial sponsor<br>L283-285
	5c	Role of study sponsor and funders, if any, in study design; collection, management, analysis, and interpretation of data; writing of the report; and the decision to submit the report for publication, including whether they will have ultimate authority over any of these activities<br>L283-285
	5d	Composition, roles, and responsibilities of the coordinating centre, steering committee, endpoint adjudication committee, data management team, and other individuals or groups overseeing the trial, if applicable (see Item 21a for data monitoring committee)<br>L95-98
**Introduction**		

For peer review only

Background and rationale	6a	Description of research question and justification for undertaking the trial, including summary of relevant studies (published and unpublished) examining benefits and harms for each intervention<br>L51-91
	6b	Explanation for choice of comparators<br>L83-86
Objectives	7	Specific objectives or hypotheses<br>L89-91
Trial design	8	Description of trial design including type of trial (eg, parallel group, crossover, factorial, single group), allocation ratio, and framework (eg, superiority, equivalence, noninferiority, exploratory)<br>L95-98
**Methods: Participants, interventions, and outcomes**		
Study setting	9	Description of study settings (eg, community clinic, academic hospital) and list of countries where data will be collected. Reference to where list of study sites can be obtained<br>L95-97
Eligibility criteria	10	Inclusion and exclusion criteria for participants. If applicable, eligibility criteria for study centres and individuals who will perform the interventions (eg, surgeons, psychotherapists)<br>L102-114
Interventions	11a	Interventions for each group with sufficient detail to allow replication, including how and when they will be administered<br>L123-160
	11b	Criteria for discontinuing or modifying allocated interventions for a given trial participant (eg, drug dose change in response to harms, participant request, or improving/worsening disease)<br>L163-173
	11c	Strategies to improve adherence to intervention protocols, and any procedures for monitoring adherence (eg, drug tablet return, laboratory tests)<br>Inapplicable
	11d	Relevant concomitant care and interventions that are permitted or prohibited during the trial

For peer review only - http://bmjopen.bmj.com/site/about/guidelines.xhtml

Outcomes	12	Primary, secondary, and other outcomes, including the specific measurement variable (eg, systolic blood pressure), analysis metric (eg, change from baseline, final value, time to event), method of aggregation (eg, median, proportion), and time point for each outcome. Explanation of the clinical relevance of chosen efficacy and harm outcomes is strongly recommended<br>L183-197
Participant timeline	13	Time schedule of enrolment, interventions (including any run-ins and washouts), assessments, and visits for participants. A schematic diagram is highly recommended (see Figure)<br>L183-184
Sample size	14	Estimated number of participants needed to achieve study objectives and how it was determined, including clinical and statistical assumptions supporting any sample size calculations\<br>L200-204
Recruitment	15	Strategies for achieving adequate participant enrolment to reach target sample size<br>L95-98

Methods: Assignment of interventions (for controlled trials)

Allocation:

Sequence generation	16a	Method of generating the allocation sequence (eg, computer-generated random numbers), and list of any factors for stratification. To reduce predictability of a random sequence, details of any planned restriction (eg, blocking) should be provided in a separate document that is unavailable to those who enrol participants or assign interventions<br>L119-125
Allocation concealment mechanism	16b	Mechanism of implementing the allocation sequence (eg, central telephone; sequentially numbered, opaque, sealed envelopes), describing any steps to conceal the sequence until interventions are assigned<br>L123-128
Implementation	16c	Who will generate the allocation sequence, who will enrol participants, and who will assign participants to interventions<br>L117-120
Blinding (masking)	17a	Who will be blinded after assignment to interventions (eg, trial participants, care providers, outcome assessors, data analysts), and how<br>Inapplicable

For peer review only - http://bmjopen.bmj.com/site/about/guidelines.xhtml

	17b	If blinded, circumstances under which unblinding is permissible, and procedure for revealing a participant's allocated intervention during the trial<br>Inapplicable

Methods: Data collection, management, and analysis

Data collection methods	18a	Plans for assessment and collection of outcome, baseline, and other trial data, including any related processes to promote data quality (eg, duplicate measurements, training of assessors) and a description of study instruments (eg, questionnaires, laboratory tests) along with their reliability and validity, if known. Reference to where data collection forms can be found, if not in the protocol<br>Please see study protocol<br>http://www.studies-obsgyn.nl/AID-study/page.asp?page_id=1562
	18b	Plans to promote participant retention and complete follow-up, including list of any outcome data to be collected for participants who discontinue or deviate from intervention protocols<br>L165-175
Data management	19	Plans for data entry, coding, security, and storage, including any related processes to promote data quality (eg, double data entry; range checks for data values). Reference to where details of data management procedures can be found, if not in the protocol<br>Please see study protocol<br>http://www.studies-obsgyn.nl/AID-study/page.asp?page_id=1562
Statistical methods	20a	Statistical methods for analysing primary and secondary outcomes. Reference to where other details of the statistical analysis plan can be found, if not in the protocol<br>L211-228
	20b	Methods for any additional analyses (eg, subgroup and adjusted analyses)<br>L218-227
	20c	Definition of analysis population relating to protocol non-adherence (eg, as randomised analysis), and any statistical methods to handle missing data (eg, multiple imputation)<br>L220-222

Methods: Monitoring

Data monitoring	21a	Composition of data monitoring committee (DMC); summary of its role and reporting structure; statement of whether it is independent from the sponsor and competing interests; and reference to where further details about its charter can be found, if not in the protocol. Alternatively, an explanation of why a DMC is not needed<br>Line 231-243

	21b	Description of any interim analyses and stopping guidelines, including who will have access to these interim results and make the final decision to terminate the trial<br>Please see study protocol<br>http://www.studies-obsgyn.nl/AID-study/page.asp?page_id=1562
Harms	22	Plans for collecting, assessing, reporting, and managing solicited and spontaneously reported adverse events and other unintended effects of trial interventions or trial conduct<br>Please see study protocol<br>http://www.studies-obsgyn.nl/AID-study/page.asp?page_id=1562
Auditing	23	Frequency and procedures for auditing trial conduct, if any, and whether the process will be independent from investigators and the sponsor<br>Please see study protocol<br>http://www.studies-obsgyn.nl/AID-study/page.asp?page_id=1562
**Ethics and dissemination**		
Research ethics approval	24	Plans for seeking research ethics committee/institutional review board (REC/IRB) approval<br>L278-282
Protocol amendments	25	Plans for communicating important protocol modifications (eg, changes to eligibility criteria, outcomes, analyses) to relevant parties (eg, investigators, REC/IRBs, trial participants, trial registries, journals, regulators)<br>Please see study protocol<br>http://www.studies-obsgyn.nl/AID-study/page.asp?page_id=1562
Consent or assent	26a	Who will obtain informed consent or assent from potential trial participants or authorised surrogates, and how (see Item 32)<br>L117-120
	26b	Additional consent provisions for collection and use of participant data and biological specimens in ancillary studies, if applicable<br>Inapplicable
Confidentiality	27	How personal information about potential and enrolled participants will be collected, shared, and maintained in order to protect confidentiality before, during, and after the trial<br>Please see study protocol<br>http://www.studies-obsgyn.nl/AID-study/page.asp?page_id=1562
Declaration of interests	28	Financial and other competing interests for principal investigators for the overall trial and each study site<br>L244-246

For peer review only

Access to data	29	Statement of who will have access to the final trial dataset, and disclosure of contractual agreements that limit such access for investigators<br>Please see study protocol<br>http://www.studies-obsgyn.nl/AID-study/page.asp?page_id=1562
Ancillary and post-trial care	30	Provisions, if any, for ancillary and post-trial care, and for compensation to those who suffer harm from trial participation<br>Please see study protocol<br>http://www.studies-obsgyn.nl/AID-study/page.asp?page_id=1562
Dissemination policy	31a	Plans for investigators and sponsor to communicate trial results to participants, healthcare professionals, the public, and other relevant groups (eg, via publication, reporting in results databases, or other data sharing arrangements), including any publication restrictions<br>L177-183
	31b	Authorship eligibility guidelines and any intended use of professional writers<br>Please see study protocol<br>http://www.studies-obsgyn.nl/AID-study/page.asp?page_id=1562
	31c	Plans, if any, for granting public access to the full protocol, participant-level dataset, and statistical code<br>Inapplicable
**Appendices**		
Informed consent materials	32	Model consent form and other related documentation given to participants and authorised surrogates<br>Please see study protocol<br>http://www.studies-obsgyn.nl/AID-study/page.asp?page_id=1562
Biological specimens	33	Plans for collection, laboratory evaluation, and storage of biological specimens for genetic or molecular analysis in the current trial and for future use in ancillary studies, if applicable<br>Inapplicable

*It is strongly recommended that this checklist be read in conjunction with the SPIRIT 2013 Explanation & Elaboration for important clarification on the items. Amendments to the protocol should be tracked and dated. The SPIRIT checklist is copyrighted by the SPIRIT Group under the Creative Commons "Attribution-NonCommercial-NoDerivs 3.0 Unported" license.

For peer review only
